# The Combined Effect of Mediterranean Shrubland Pasture and the Dietary Administration of Sage By-Products on the Antioxidant Status of Segureña Ewes and Lambs

**DOI:** 10.3390/antiox9100938

**Published:** 2020-09-30

**Authors:** María J. Jordán, Cristina Martínez-Conesa, Sancho Bañón, Julio Otal, María Quílez, Inmaculada García-Aledo, Pascual Romero-Espinar, Pedro Sánchez-Gómez

**Affiliations:** 1 Research Group on Rainfed Crops for the Rural Development, Murcia Institute of Agri-Food Research and Development (IMIDA), c/Mayor s/n, 30150 La Alberca Murcia, Spain; cristina.martinez4@carm.es (C.M.-C.); maria.quilez@carm.es (M.Q.); inmaculada.garcia15@carm.es (I.G.-A.); pascual.romero2@carm.es (P.R.-E.); 2 Department of Food Technology, Veterinary Faculty University of Murcia, 30100 Murcia, Spain; sanchoba@um.es; 3 Department of Animal Production, Faculty of Veterinary Science, University of Murcia, 30100 Murcia, Spain; juotal@um.es; 4 Department of Plant Biology (Botany), Faculty of Biology, University of Murcia, Campus de Espinardo, E-30100 Murcia, Spain; psgomez@um.es

**Keywords:** Segureña ewe, blood antioxidant enzymes, plasma antioxidant capacity, Mediterranean shrublands, *Salvia lavandulifolia* by-products, meat antioxidant capacity

## Abstract

The objective of this study was to determine if the combined effect of ewe grazing and the incorporation of sage by-products in the lamb feed enhances the Segureña ewe and lamb antioxidant status. For that, the endogenous (related to blood antioxidant enzymes) and exogenous (plasma antioxidant activity) antioxidant systems of ewes and lambs were studied at the physiological stages of lactation, after weaning and at the end of the fattening period. Major results indicated that grazing improved the enzymatic antioxidant defense of ewes during the stressful period of lamb weaning, and also, the antioxidant status of the ewe plasma at both physiological stages: lactation and after lamb weaning. With regards to the lambs, ewe grazing stimulated the antioxidant enzymatic defense during lactation, but not the antioxidant capacity of the plasma. At the end of the fattening period, the inclusion of sage by-products in the lamb diet (10% *w*/*w*) enhanced both the enzymatic cascade and antioxidant capacity of the lamb plasma. The antioxidant capacity of the lamb meat was also studied and the benefits of sage were detected in samples from the *deltoideus* muscle. Thus, Mediterranean grazing stimulated the antioxidant defense in ewes, while the inclusion of sage by-products in the lamb diets enhanced the antioxidant status of both blood and meat.

## 1. Introduction

The Segureña sheep breed constitutes one of the pillars in the economic activities of the Southwest Mediterranean rural areas. The traditional system of Segureña breed sheep production in Southeastern Spain is based on allowing the sheep to graze all year round, taking advantage of the natural resources available. In this area, the pasture resources are basically the by-products of cereal crops (straw, fallow lands and poor harvests) and the shrubland communities of the forest area [1]. However, lambs are raised under an intensive regime, and receive the benefits of pasture only through their mothers during gestation and lactation periods. Previous studies in Segureña and Merina sheep, conducted by Jordán et al. [2] and Morán et al. [3] on the dietary introduction of the by-product remaining after the distillation of rosemary and thyme, (species that are usually present in the mountain pastures of the Mediterranean area) demonstrated the transfer of beneficial compounds to the meat and detoxification organs of these animals. These compounds and their metabolites cause an improvement in the meat oxidative stability and, at the same time, provide an important benefit to animal health and welfare [4]. Oxidative stress is the consequence of an imbalance between the formation of oxidants and their detoxification by the antioxidant system, and it has been implicated in numerous disease processes, including sepsis, mastitis, acidosis, ketosis, enteritis, pneumonia and joint diseases [5]. Ognik et al. reported that the antioxidant potential of lamb blood increases with age and is affected by suckling or not [6]. Weaning, a stressful process, is followed by the adaptation of the animal to a new feeding pattern, leading to an increased risk of oxidative stress. Therefore, diets enriched with natural antioxidants may be used to alleviate the negative effects of oxidative stress by increasing the antioxidant potential of ruminants [7,8]. In line with this, aromatic and medicinal plant by-products are known to be important sources of polyphenolic components with high antioxidant power. Moreover, in the South-East of Spain, where the Segureña breed is raised, the cultivation and extraction of essential oils from these aromatic plants, mainly *Salvia lavandulifolia* Vahl., represent a supplement to the incomes of the rural population. As a result of this activity, a significant volume of by-products from the distillation process is produced every season. Distilled sage by-product is an important source of polyphenols with a high antioxidant power [9].

As stated in the above paragraphs, the scientific literature related to the benefits of ewe grazing mentions the increased antioxidant potential for suckling lambs. The alleviation of oxidative stress in lambs after the introduction of antioxidant fractions during the fattening stage has also been described. However, to the best of our knowledge, the accumulative effect of sheep grazing plus antioxidant supplements during the fattening period has not been reported before in relation to the alleviation of oxidative stress. The main goal of the present study was to investigate whether the combined effect of providing ewes with autumn-winter grazing and the introduction of sage by-products in the fattening lambs’ diet improves their antioxidant status.

## 2. Materials and Methods

### 2.1. Animals and Diets

The Segureña ewes were from a commercial farm in the Segura mountain range in the North-West of the province of Murcia (Spain). The experiment involved two replications (20 days between repetitions) and was carried out between August 2015 and February 2016, as previously described by Mateo et al. [10].

Briefly, a total of 64 pregnant ewes (32 per replication) were randomly selected, on the bases of their age (3.2 years old) and body condition (2.63 ± 0.15, according to the method previously described by Russell et al. [11], and allocated into two homogeneous groups (16 ewes per batch) corresponding to two different rearing systems (intensive and semiextensive). In the intensive group (zero grazing system), ewes were fed indoors in individual pens, along with their lambs, on barley grain and lucerne pellets, 1:1 (1.2 kg per ewe/day). The semiextensive group grazed about 8 h daily, on a local extension of 480 hectares of Mediterranean shrubland, and spent the night indoors, together with the lambs, in a collective pen. Both rearing systems allowed the ewes to feed on oat and barley straw in pens (about 200 g per ewe and day). The animals were kept under these conditions during pregnancy (150 days) and until weaning of lambs (45 days).

In order to describe the vegetation grazed by the ewes, animals were tracked throughout the mountain range on alternate days during 1 week, and samples of the species that were bitten at least twice by the sheep were collected for analysis. Based on the study of the area by aerial photography and in situ coverage sampling, bare soil was estimated to represent 37.5% of the surface area. The trees consisted of specimens with an average diameter of 6.3 ± 2.83 m, belonging to the species *Pinus* spp. (75%) and *Quercus rotundifolia* (25%). The coverage of shrub species was 29.2 ± 17.0% and that of herbaceous species was 8.3 ± 6.29%.

Lambs began to be born in December 2015 and the time between the first birth and the last birth was 45 days. The lactation periods lasted from December 2015 to January 2016 (first replication) and from January to February 2016 (second replication). All the lambs were weaned when they reached a live weight of 13 ± 1 kg.

A total of 4 groups of 10 weaned lambs (10 lambs × 2 ewe rearing systems × 2 replications) were randomly assigned to two experimental diets: control, based on a grain-concentrate feed (Table S1) and distilled sage, in which the lambs received the same feed but with 100 g of sage by-products per kg of feed. All the lambs were fed ad libitum, in individual pens, for 80 ± 5 days until they reached a live weight of 25 ± 2 kg, following the practices recommended by Segureña farmers. The average daily feed intake for weaned lambs was 693.6 ± 54.0 g per animal. There were no significant differences (*p* > 0.05) in the average feed intake between the lamb groups.

All handling practices followed the recommendations of the European Council Directive 2010/63/EU for the protection of animals used for experimental and other scientific purposes.

In total, 80 fattened lambs were available for this study, with the same number of male and female lambs per batch and experimental diet. The research project was approved by the 145/2015 report of the Ethical Committee on Animal Research (CEEA) of the University of Murcia.

### 2.2. Polyphenolic Content of Ewe and Lamb Diets

To extract the polyphenols, dried samples (0.5 g) from the aerial parts of individual plants were extracted with 150 mL of methanol. The qualitative and quantitative analyses of the polyphenolic components were carried out by liquid chromatography coupled to a photodiode array UV/Vis detector, following the method described by Jordán et al. [12]. Identification of the phenolic components was made by comparison of retention times and spectra with those of commercial standard compounds. For quantification, linear regression models were determined using standard dilution techniques. The phenolic compound contents were expressed in milligrams per gram of dry plant material.

### 2.3. Antioxidant Activity

#### 2.3.1. Total Phenolic and Tannin Content

Colorimetric quantifications of the total phenolic and tannin contents were made according to the methods described by Singleton and Rossi [13] and by Makkar et al. [14], respectively. For the total phenolic content, methanolic extract (15 µL) was added to 1185 µL of distilled water, 75 µL of Folin-Ciocalteau reagent and 225 µL of sodium carbonate (20%). The absorbance of the resulting blue-colored solution was measured at 765 nm, at 25 °C, with a Shimadzu (UV-2401 PC, Shimadzu Coorporation, Kyoto, Japan) spectrophotometer. Quantitative measurements were performed, based on a standard calibration curve of concentrations ranging from 25 to 300 mg/L of tannic acid equivalents (TAEs). The results were expressed in milligrams of TAEs per gram of dry plant material.

To determine the tannin content, 1 mL of the methanolic extract was added to 100 mg of polyvinylpyrrolidone (PVP), diluted with 1 mL of distilled water (pH = 3) and mixed vigorously. After this, the solution was centrifuged at 3000× *g* for 10 min and the supernatant was collected. The total phenolic content in this supernatant (400 µL) was determined as described above.

#### 2.3.2. Ferric Reducing Antioxidant Power (FRAP)

The ability to reduce ferric ions was measured using the method described by Benzie and Strain [15]. Antioxidant compounds are able to reduce ferric to ferrous-TPTZ, which develops a blue color with an absorption maximum at 593 nm. The FRAP reagent was freshly prepared from 300 mM acetate buffer, pH 3.6, 10 mM TPTZ made up in 40 mM HCl and 20 mM FeCl_3_·6H_2_O solution. All three solutions were mixed together in the ratio of 10:1:1 (*v*/*v*/*v*). An aliquot of 40 μL of each sample (with appropriate dilution) was added to 1.2 mL of FRAP reagent. Readings at maximum absorption (593 nm) were taken after 2 min of incubation at 37 °C. Measurements were performed in triplicate. Fresh working solutions of known Fe (II) concentrations (FeSO_4_·7H_2_O of 0–2 mM) were used for calibration, and results were expressed as mol of Fe (II) equivalent per gram of dry plant.

#### 2.3.3. DPPH^●^ Radical-Scavenging Activity

The ability of the methanolic extracts to scavenge DPPH^●^ free radicals was determined according to the method described by Brand-Williams et al. [16]. Briefly, 500 μL of methanolic extracts at different concentrations (from 2 to 15 μL/mL) were added to 1 mL of DPPH^●^ methanolic solution (0.1 mM). Decoloration was measured using a Shimadzu (UV-2401PC, Shimadzu Coorporation, Kyoto, Japan) spectrophotometer at 517 nm after incubation for 20 min at room temperature in the dark. Absorbance was measured against a blank of 500 μL of sample plus 1 mL of methanol. Measurements were performed in triplicate. The percentage activity for the DPPH^●^ was calculated according to equation below:

% decoloration = [1 − (Absorbance sample/Absorbance control)] × 100(1)

The results were expressed as the inhibitory concentration of the extract necessary to decrease DPPH^●^ absorbance by 50% (IC_50_). Concentrations were expressed in micrograms of dry plant per milliliter of methanol.

### 2.4. Blood and Plasma Samples

Blood samples from ewes, and suckling and fattened lambs were obtained by jugular venepuncture. In both ewes and lambs at the lactation stage (day 20), and 2 days after weaning (day 45), and in lambs before slaughtering (day 80), samples (10 mL) were collected in Li-heparinized evacuated tubes (Belliver Industrial Estate, Plymouth, UK), which were immediately placed in a cool-box. Within 2 h after bleeding, total blood (500 µL) was preserved for determination of the antioxidant enzymes catalase, superoxide dismutase and glutathione peroxidase. A total of 5 mL of blood was used to extract the plasma, centrifuging the tubes at 2500× *g* for 10 min at 4 °C. The plasma was acidified (1:11) with acetic acid (10 mM) and kept at −80 °C until analysis. The remaining 450 µL of whole blood was put into Li-heparin treated tubes and stored at 4 °C until analysis of the blood cell count. For the determination of the hemoglobin content in ewes and lambs an ADVIA 120 hematology analyzer (Siemens Healthineers, Madrid, Spain) was used.

In order to obtain the enzymatic extract, 3.5 mL of distilled cool water, plus 3.5 mL of ethanol and 1.25 mL of chloroform were added to 0.5 mL of whole blood. The mixture was shaken vigorously for 2 min, and cell membranes were removed by centrifugation at 18,000× *g* for 1 h at 4 °C. The hemolysates were then used to determine the antioxidant enzyme levels.

### 2.5. Antioxidant Enzymes

#### 2.5.1. Catalase, Superoxide Dismutase and Glutathione Peroxidase

Catalase (CAT) activity was measured according to the method described by Aebi [17]. Thus, 2 mL of enzymatic extract was placed in a quartz cuvette (1 cm path length) and 1 mL of hydrogen peroxide 30 mM in phosphate buffer (pH 7.0; 0.05 M) was added. The decrease in H_2_O_2_ was monitored at 240 nm for 30 s in a Shimadzu spectrophotometer (UV-2401PC, Shimadzu Coorporation, Kyoto, Japan). The rate of disappearance of H_2_O_2_ is directly proportional to CAT activity, and was expressed as enzymatic unit (EU) per g^−1^ of hemoglobin. In total, 1 U of catalase activity was defined as the amount of extract needed to decompose 1 µmol of H_2_O_2_ per minute.

#### 2.5.2. Superoxide Dismutase

Superoxide dismutase (SOD) activity was determined according to the protocol published by Marklund and Marklund [18]. This antioxidant enzyme reduces superoxide anion radicals to H_2_O_2_ and oxygen. Based on this, SOD activity was measured by the inhibition of pyrogallol oxidation. Superoxide radicals (O_2_^●−^) were obtained by the chemical autoxidation of pyrogallol at pH 8.2. For the detection of these radicals, nitro blue tetrazolium (NBT), a yellow colorant, was used. In the presence of O_2_^●−^, NBT is chemically reduced to NBT formazan, which can be measured spectrophotometrically at 540 nm. The inhibition of NBT reduction was measured by this method. A total of 500 µL of tris-cacodylic buffer (pH = 8.2), plus 100 µL of Triton X-100 (16%) and 250 µL of NBT 0.98 mM were added to 250 µL of enzymatic extract. A control tube was also prepared by substituting the enzymatic extract with pure distilled water. The reaction was initiated by adding 10 µL 0.9 mM pyrogallol to both cuvettes, and a first reading was taken at 540 nm. Next, both cuvettes were incubated at 37 °C for 5 min and a stopper solution (formic buffer 2M pH = 3.5 in Triton X-100 at 16%) was added. The increment in absorbance was measured, and the percentage of inhibition was calculated by the equation:

%Inhibition = [(∆ Acontrol − ∆ Asample)/∆ Acontrol] × 100(2)

One unit of SOD activity was defined as the amount of extract needed to inhibit NBT reduction by 50%. The SOD activity was expressed as enzymatic unit (EU) per g^−1^ of hemoglobin.

#### 2.5.3. Glutathione Peroxidase

The antioxidant activity of glutathione peroxidase (GSH-Px) was determined following the protocol previously published by Paglia and Valentine [19]. In this procedure, H_2_O_2_ was used as a substrate, considering the capacity of GSH-Px to reduce hydrogen peroxide to water. The technique is based on the oxidation of reduced glutathione (GSH) to oxidized glutathione (GSSG) catalyzed by GSH-Px, which is then coupled to the recycling of GSSG back to GSH utilizing glutathione reductase (GR) and β-nicotinamide adenine dinucleotide phosphate (NADPH). The action of GR was monitored by following the disappearance of the cosubstrate NADPH at 340 nm. Thus, to determine the GSH-Px activity, the amount of NADPH used to reduce GSSG to GSH was measured spectrophotometrically. For this procedure, 1325 µL of reaction solvent containing 1.13 mM GSH, 0.57 mM EDTA, 1.13 mM NaN_3_ and 1.7 units of GSH reductase in 100mL phosphate buffer (pH 7.0; 50mM disodium phosphate dodecahydrate (Na_2_HPO_4_·12H_2_O) and KH_2_PO_4_ in distilled water), 13 µL NADPH solution (17.3 mM) and 10 µL H_2_O_2_ solution were added to 150 µL of enzymatic extract in a cuvette. The absorbance was monitored at 340 nm for 360 s. The extinction coefficient of 6220 µL µmol^−1^ cm^−1^ for NADPH at 25 °C was used for the calculation. GSH-Px activity was expressed as enzymatic unit (EU) per gram of hemoglobin^−1^.

### 2.6. Plasma Antioxidant Capacity. ABTS^●+^ Radical Cation Decoloration Assay

To determine the antioxidant activity of the ewe and lamb plasma, a modified version of the method described by Cerdá et al. [20] was followed. In this procedure, 5 mL of acidified plasma was applied to a polymeric resin, Chromabond HR-P (polystyrene-divinylbenzene) (Macherey-Nagel, Duren, Germany), using methanol as an extracting solvent. Methanolic extracts (25 mL) were dried at 40 °C under vacuum conditions in an evaporator system (Syncore Polyvap R-96, Buchi, Barcelona, Spain). The residue was dissolved in methanol and brought to 5 mL.

The Trolox equivalent antioxidant capacity (TEAC) assay was used for the determination of the plasma methanolic extract antioxidant activity, according to the method described by Re et al. [21].

An aliquot (15 μL) of each sample (with appropriate dilution), or a Trolox standard, was mixed with the working solution (1.5 mL) of ABTS^●+^, and the decrease in absorbance was measured after 6 min at 734 nm using a Shimadzu (UV-2401PC, Shimadzu Coorporation, Kyoto, Japan) spectrophotometer. The measurements were performed in triplicate. The results were expressed in terms of mmol of Trolox equivalents per milliliter of plasma.

### 2.7. Antioxidant Status of Meat Samples

After the rearing period, all the animals were slaughtered in a local abattoir according to EC Regulations. The carcasses were chilled at 2 °C for 48 h in a cooling room. Fresh samples of meat from the loin (*longissimus dorsi* muscle), the front legs (*Musculus deltoideus*) and the abdominal wall (*Musculus obliquus externus abdominis*) were removed from the carcasses, cut into pieces, vacuum-packed and stored at 80 °C prior to analysis.

Chopped meat from the different muscles were lyophilized (VirTis, 6K BTEL-85 freeze drier, Ucoa-Erloss, Madrid, Spain) and kept in a dry atmosphere prior to analysis. Dried samples (1.5 g) were extracted using 150 mL of methanol in a Soxhlet extractor, as previously described for the polyphenolic extraction in vegetal matter. For the determination of antioxidant status, the total phenolic content, ferric reducing antioxidant power (FRAP) and DPPH^•^ radical-scavenging activity were determined by following the protocols described in the above paragraphs but with the corresponding modifications.

For the FRAP determination 0.04 mL of meat methanolic extract and 0.12 mL of deionized water were added 1.2 mL of reagent. Readings at maximum absorption (593 nm) were taken every 15 s, and the reaction was monitored for up to 60 min. The endogenous Fe (II) content of meat (FRAP_o_) was determined with a TPTZ/HCl solution without ferric chloride in the reaction mixture. Results were expressed as mmol Fe (II) equivalent per gram of fresh meat. For the radical scavenging activity, meat methanolic extracts (500 µl) at different concentrations (25 to 400 µL/mL) were added to 1 mL of methanolic DPPH^●^ solution (0.1 mM). The estimated time of reaction (20 min) was determined by considering the reduction in absorbance at 517 nm (monitored every 5 min), until the reaction curve reached a plateau. The results were expressed as the inhibitory concentration of the extract necessary to decrease DPPH^●^ absorbance by 50% (IC_50_). Concentrations were expressed in milligrams of fresh meat per milliliter of methanol.

### 2.8. Statistical Analysis

#### 2.8.1. Ewes and Lambs (Lactation and Weaning Periods) Statistical Model

Individual ewes and lambs were the experimental unit. To compare the groups, analysis of variance (ANOVA) was used. Means were compared by least significant differences (LSD), and a probability of *p* < 0.05 was adopted as the criterion for significant differences.

#### 2.8.2. Lambs Fattening Period Statistical Model

A factorial model was designed completely at random. The effects of the mother rearing system and the dietary treatments on the dependent variables were determined by two-way ANOVA. A least significant difference means test was used to compare the least square means, which were considered to be statistically different when *p* < 0.05.

Results were presented as means and standard error of the means. Statistical analysis of the blood and meat values was conducted using the Excel (v.15.0, Microsoft Corporation, Redmond, Washington, DC, USA) and IBM SPSS Statistic 25 programs (v.25.0, IBM, Armonk, New York, NY, USA).

## 3. Results

### 3.1. Polyphenolic Content and Antioxidant Activity of Ewe and Lamb Diets

The plants grazed by sheep in the Mediterranean shrublands are described in Table 1. Among the pasture species present in the area grazed by the sheep, shrubs were the most abundant, representing 60% of the total identified.

The polyphenolic profiles and antioxidant activities of the grazed species are shown in Table 2, Table 3 and Table 4. In regard to the arborescent species, *Quercus rotundifolia* (oak) was one of the foraged species with the highest antioxidant activity (Table 4). The major polyphenols quantified in this species were catechin, gallic acid, rutin hydrate and rosmarinic acid. Regarding the herbaceous species, *Helianthemum cinereum*, which contained gallic acid, luteolin-7-*O*-glucoside and kaempferol as the major polyphenolic components quantified, exhibited a powerful antioxidant activity, comparable to that of oak. Shrub species, including the genus *Juniperus* (*oxycedrus*, *phoenicea* and *thurifera*), showed high catechin and rutin hydrate contents. These species are also included among the grazed plants with the most powerful antioxidant capacities. In *Lithodora fruticosa*, components with a high antioxidant activity were found as part of the major polyphenolic profile, including rosmarinic acid, salvianolic acid B, rutin hydrate and salvianolic acid. For plants belonging to the Labiatae family, the species included in Table 3 are rich in phenolic components with a marked antioxidant capacity; among them, it is interesting to highlight the presence of carnosic acid in rosemary, and rosmarinic acid in salvia and thyme. All these species exhibited similar antioxidant activities, close to those detected for *L. fruticosa*; rosmarinic acid along with other phenolic acids, as mentioned in the above paragraph, were responsible for this high antioxidant power (Table 4).

The quantitative polyphenolic profile of the *Salvia lavandulifolia* by-products (added to the lamb feed at 10%) is also reported in Table 5. The major polyphenolic components identified were the phenolic acids, rosmarinic and salvianolic, along with the flavonoids salvigenin, genkwanin, cirsimaritin, luteolin and eriodyctiol. As expected, these components were present at lower concentrations in the by-products, compared to the wild plants, as a consequence of the distillation processes they had undergone.

### 3.2. Enzymes Related to Oxidative Stress

The extent to which cellular oxidative stress was alleviated served as an indicator of improved animal health and welfare status, and was measured in ewe and lamb blood at different physiological stages (Table 6). During the lactation period, the antioxidant enzymatic activities in ewe blood did not show statistically significant differences between the animals from the two rearing systems. However, after weaning, a positive effect on the activities of these antioxidant enzymes was noticed in animals that had grazed, which had a significantly lower value for the antioxidant enzymatic cascade (*p* = 0.04). The results concerning the effect of the mother’s grazing on the enzymatic antioxidant system of lamb blood verified the beneficial effect of pasture intake during lamb lactation on the synergistic action of these three enzymes (*p* = 0.02). After weaning, the previous beneficial effect of ewe grazing was not strong enough to maintain lower values in the lamb blood enzymatic antioxidant cascade, compared to the values found in lamb blood from the intensive ewe rearing system. Following these studies, the effect of the ewe rearing system on the lamb blood antioxidant system at the end of the fattening period was also determined. To see if they improved the endogenous antioxidant defense of the lambs, distillation by-products from *S. lavandulifolia* were added to the animal diet. The results (Table 6) show that the beneficial effect of sheep grazing during gestation and lactation was evident at this time, regardless of whether the diet was supplemented with sage by-products. By contrast, in the intensive system, which lacked the benefits of the mother’s grazing on pasture, the inclusion of sage enhanced the alleviation of oxidative stress to levels close to those associated with semiextensive production (*p* = 0.04).

### 3.3. Plasma Antioxidant Activity

The results concerning the plasma antioxidant capacity are shown in Table 7. In ewes, the effect was positive during the lactation period (*p* < 0.01) and after weaning (*p* = 0.04). However, in lambs, the plasma antioxidant power was improved to a statistically significant extent only at the end of the fattening period in animals fed the sage by-products (*p* < 0.01).

### 3.4. Antioxidant Capacity of Lamb Meat

The antioxidant activity, measured by the ferric reducing antioxidant power, the antiradical activity against DPPH^●^ radical and the total polyphenolic content were determined in three lamb muscles (*longissimus dorsi*, *deltoideus*, and *abdominis*), which were chosen considering the different metabolic processes existing in these tissues. See Table 8.

In the case of the lamb loin, the results confirmed the positive effect of the grazing on Mediterranean shrublands, since the meat from lambs of ewes reared outdoors exhibited higher antioxidant capacity regardless of whether sage by-product had been added to their diets. In the meat from the front legs, ewe grazing was also seen to have a positive benefit on the antioxidant status of these meat samples; in this case, the introduction of sage by-product in the lamb feed increased the antioxidant status of the meat samples. By contrast, in the muscles from the abdominal wall, whose intramuscular fat content was higher, there was no beneficial effect of either factor—ewe rearing system or the introduction of sage in the diet.

## 4. Discussion

It is known that the beneficial effects of plant secondary metabolites, especially polyphenolic components, on livestock are associated with their antioxidant functions [22]. However, according to Poutaraud et al. [23], there is no accurate information available concerning the dietary intake of polyphenols for herbivore husbandry. This fact explains the interest in assessing the major polyphenolic components synthesized by the plants normally grazed by sheep in Mediterranean shrublands during the autumn. *H. cinereum* and *Q. rotundifolia* were the species with the greatest potential benefits. It is also interesting to highlight the high proportion of tannins quantified in this latter arboreal plant. Although these polyphenolic polymers have often been classified as antinutritional factors, Piluzza et al. [24] described them as molecules with beneficial effects for animals and the environment. Plants from the genus *Juniperus* also feature here among the most active antioxidant shrubs. However, in the scientific literature, there is some controversy about whether the introduction of *Juniperus* spp. in ruminant feeding can be beneficial or not. On the one hand, Achak et al. [25] showed that *Juniperus* spp. contain terpenes and other secondary metabolites, like tannins, which restrict their intake and negatively affect the health of goats. On the other, the positive effects of these shrubs on ruminant nutrition were defined by Stewart et al. [26]. In this case, the presence of ground redberry juniper in the feed of gestating ewes, in the pre and postpartum physiological stages, did not have any effect on the preweaning performance of the progeny. With regards to the Labiatae family, as stated in the results section, the species grazed by sheep are also among the most interesting plants. According to Alagawany et al. [27], the supplementation of livestock rations with herbs containing rosmarinic acid, as a natural feed additive, gives promising results as a way of promoting growth, productive and reproductive performance, feed utilization, fertility, antioxidant status and immunological indices. The presence of the diterpenes carnosic acid and carnosol in rosemary also justifies the high antioxidant potential of this plant, regarding the positive effect of its inclusion in the diet on the antioxidant status of lamb [2]. Thus, these species, which are rich in the mentioned polyphenols, when grazed by sheep could represent interesting antioxidant sources, and their intake by livestock might be an efficient mean to enhance the endogenous and exogenous antioxidant systems.

Related to this, it is known that an increase in energetic requirements in stress situations, as occurs during lactation and after lamb weaning, stimulates the oxidative metabolism, which induces the production of free radicals [28]. Endogenous antioxidants, which include the enzymes SOD, CAT and GSH-Px, represent the main form of intracellular antioxidant defense [29]. According to Amstad et al. [30], the balance between the SOD, CAT and GSH-Px activities is a more accurate indicator of the oxidative damage than the individual action of each enzyme on its own. Hence, when the ratio among these three activities is low, lower concentrations of reactive oxygen species will appear inside the cell, and they will be easily reduced by the exogenous antioxidant system [31]. On the basis of these activities, pasture intake lowered the oxidative stress of the ewes after lamb weaning, and of the lambs during lactation. In line with these results, Calderón-Montano et al. [32] revealed that dietary antioxidants may decrease superoxide anion, hydroxyl radical and peroxynitrite levels, inhibit the activities of enzymes that generate reactive oxygen species (ROS), such as xanthine oxidase and increase the expression of antioxidant enzymes such as SOD or CAT, which could explain the results of the present study. Differences between enzymatic levels during lactation and after the weaning stage could be attributed to the different levels of stress suffered by ewes. Grazing animals are separated from their lambs for eight hours a day, which, in the early lactation stage, may generate stress, inducing an increase in ROS production. It is known that high levels of these oxidative species reduce the activity of the CAT and GSH-Px antioxidant enzymes [33]. The beneficial effect of ewe grazing on the lamb blood enzymatic antioxidant activity detected during lactation can be explained by considering the previous studies carried out in our facilities, in which a transfer of polyphenols during the lactation period to the suckling goat kid plasma was demonstrated [34]. Later, Ognik et al. [6] confirmed that dams’ milk, which is rich in antioxidants, helps maintain the balance between free radical production and detoxification in young ruminants. These effects were also observed in lambs at the end of the fattening period, since the outdoor ewe rearing system improved the antioxidant enzymatic defense of the animals. This positive effect could be related to the transfer of antioxidant polyphenols to different lamb tissues during gestation and lactation; they would remain in the bloodstream and improve the antioxidant defense of these ruminants. The absence of this positive effect in the blood of lambs obtained from ewes of the indoor system confirms this view. In this case, the introduction of sage by-products, rich in antioxidants, was necessary to maintain the balance between the activities of the antioxidant enzymes close to that of lambs obtained from ewes of the outdoor rearing system. The introduction of antioxidants (lycopene) in the diet of lambs and its effect on the antioxidant status was studied by Jiang et al. [7]. These authors observed decreases in the activities of the antioxidant enzymes (GSH-Px and SOD), which suggested a poorer oxidative status in lambs.

An increase in the antioxidant capacity of ewe plasma was also determined, which supports the premise that daily grazing reinforces the antioxidant defense of the animals, improving their wellbeing and productivity [35]. In lambs, the antioxidant capacity of plasma was expected to increase with the age of the animals [6], and also in the lambs being reared with their mothers under grazing conditions. However, contrary to this, our results suggest that ewe grazing in the Mediterranean shrublands had no significant effect on the antioxidant status of lamb plasma. Such a beneficial effect was only achieved, at the end of the fattening period, by the introduction of sage by-products in the lamb feed. Thus, in agreement with Ognik et al. [36] and Jiang et al. [7], an increase in the antioxidant potential of ruminants can be attained by dietary means. The presence of these polyphenolic components in the bloodstream could entail their deposition in the animal tissues [2,37]. For this reason, the transfer of these components to muscles with different metabolic activities was studied by considering any increase in their antioxidant activities. According to the results obtained, even the fact that lambs have doubled their weight (from weaning to the end of the fattening period), the polyphenols deposition through lactation was still active. Previous studies carried out by Mateo et al. [10], which formed part of the present research project, showed that regardless of the ewe rearing system (indoors vs. grazing), the introduction of sage by-products in the lamb diet did not improve the antioxidant stability of lamb loin under retail conditions. Hence, it can be concluded that there was a positive transference of metabolites with antioxidant activity to the lamb tissues but they did not have the power to maintain the oxidative stability of the meat under retail conditions. Differences in the antioxidant status depending on the muscle under study confirm the need to study different tissues (from different muscles) in order to assess the real effect of ewe rearing system and the diet on the antioxidant status of lamb meat.

## 5. Conclusions

In Mediterranean shrublands the plant species commonly grazed by ewes represent an important source of antioxidant components and the intake of these plants by Segureña sheep enhances the antioxidant defenses of both sheep and suckling lambs. More specifically, pasture intake reinforced the enzymatic antioxidant defense of ewes and their plasma antioxidant capacity and, at the same time, stimulated the suckling lamb antioxidant enzymatic cascade, but not the antioxidant capacity of the plasma. At the end of the fattening period, the beneficial effect of maternal grazing was evident in the antioxidant defense system, regardless of any dietary supplementation with distilled sage leaves. Contrary to this, in the indoors system, which lacked the benefits associated with grazing, the inclusion of sage by-products in the lamb diets enhanced the antioxidant enzymatic system and the antioxidant capacity of lamb plasma. The improvement in the resulting lamb meat antioxidant status varied depending on the muscle, but could be achieved by the combination of ewe grazing plus supplementation with distilled sage leaves.

## Figures and Tables

**Table 1:** List of plants grazed by sheep in the Mediterranean shrubland.

Family	Species
*Fagaceae*	*Quercus rotundifolia*
*Pinaceae*	*Pinus halepensis*
	*Pinus nigra*
*Cupressaceae*	*Juniperus oxycedrus*
	*Juniperus phoenicea*
	*Juniperus thurifera*
*Lamiaceae*	*Ballota hirsuta*
	*Genista scorpius*
	*Phlomis lychnitis*
	*Rosmarinus officinalis*
	*Salvia lavandulifolia*
	*Sideritis leucantha*
	*Thymus vulgaris*
*Apiaceae*	*Bupleurum fruticosum*
*Boraginaceae*	*Lithodora fruticosa*
*Caryophyllaceae*	*Paronychia argentea*
*Cistaceae*	*Helianthemum cinereum*
*Asteraceae*	*Atractylis humilis*

**Table 2:** Major polyphenolic components quantified in the species grazed by sheep (mg/g of dry plant).

**Components**	**Arborescent Species**		
	*Q. rotundifolia*	*P. halepensis*	*P. nigra*
Catechin	7.4 ± 4.72	1.3 ± 0.18	1.9 ± 1.06
Gallic acid	0.9 ± 0.60	--	--
Rutin hydrate	0.6 ± 0.11	--	--
Rosmarinic acid	0.4 ± 0.32	--	--
	**Herbal Species**		
	*H. cinereum*	*P. argentea*	*A. humilis*
Gallic acid	1.2 ± 0.90	--	--
Luteolin-7-*O*-glucoside	1.7 ± 1.23	--	--
Kaempferol	0.1 ± 0.05	--	--
Rutin hydrate	--	0.3 ± 0.20	0.2 ± 0.15
Naringenin	--	0.2 ± 0.18	0.1 ± 0.06
4-*O*-caffeoylquinic acid	--	0.1 ± 0.07	0.1 ± 0.08
Luteolin	--	0.4 ± 0.36	0.04 ± 0.02
Apigenin	--	0.2 ± 0.12	0.1 ± 0.09
Chrysoeriol	--	0.3 ± 0.14	0.1 ± 0.04
	**Shrubs**		
	*J. oxycedrus*	*J. phoenicea*	*J. thurifera*
Catechin	8.6 ± 3.2	8.0 ± 2.9	1.0 ± 0.92
Rutin hydrate	8.3 ± 1.30	4.3 ± 1.6	3.4 ± 2.9
	*L. fruticosa*		*B. fruticosum*
Rosmarinic acid	3.3 ± 2.65		--
Salvianolic acid B	1.1 ± 0.63		--
Rutin hydrate	0.5 ± 0.1		5.6 ± 3.5
Salvianolic acid	0.2 ± 0.15		--
4-*O*-caffeoylquinic acid	--		0.1 ± 0.23
	*G. scorpius*	*B. hirsuta*	*S. leucantha*
Luteolin-7-*O*-glucoside	7.6 ± 3.5	0.4 ± 0.03	--
Luteolin	0.5 ± 0.4	0.9 ± 0.75	--
4-*O*-caffeoylquinic acid	--	--	1.5 ± 0.96
Protocatechuic acid	--	--	1.4 ± 1.23
Diosmetin	--	--	0.2 ± 0.05
Cirsimaritin	--	--	0.1 ± 0.02

Values are means ± SD of three independent replicates from three different samples of each species. -- not detected.

**Table 3:** Major polyphenolic compounds quantified in the shrub pasture species belonging to the Labiatae family (mg/g of dry plant).

Components	*Ph. lychnitis*	*R. officinalis*	*S. lavandulifolia*	*Th. vulgaris*
Salvianolic acid	0.1 ± 0.03	0.2 ± 0.01	0.4 ± 0.02	0.2 ± 0.05
Chlorogenic acid	--	0.3 ± 0.31	--	--
Protocatechuic acid	0.1 ± 0.06	--	--	--
Neochlorogenic acid	0.1 ± 0.06	--	--	--
4-*O*-Caffeoylquinic acid	2.0 ± 1.83	0.3 ± 0.18	--	--
Caffeic acid	--	0.2 ± 0.02	0.3 ± 0.05	--
Luteolin-7-*O*-glucoside	0.3 ± 0.20	--	1.5 ± 0.37	2.0 ± 0.01
Hesperidin	--	2.7 ± 1.02	--	--
Rosmarinic acid	1.4 ± 0.96	8.6 ± 2.54	15.3 ± 1.33	8.3 ± 0.95
Luteolin	0.1 ± 0.01	0.1 ± 0.01	0.2 ± 0.02	0.3 ± 0.30
Apigenin	0.1 ± 0.09	0.1 ± 0.06	0.1 ± 0.02	0.1 ± 0.05
Hispidulin	--	0.1 ± 0.01	0.1 ± 0.04	--
Diosmetin	--	0.1 ± 0.01	0.1 ± 0.05	0.1 ± 0.09
Cirsimaritin	0.1 ± 0.02	0.2 ± 0.03	0.6 ± 0.13	0.3 ± 0.22
Genkwanin	--	0.5 ± 0.08	0.1 ± 0.02	0.2 ± 0.03
Salvigenin	--	--	0.6 ± 0.09	--
Carnosol	--	9.8 ± 6.14	--	--
Carnosic acid	--	24.9 ± 11.3	--	--

Values are means ± SD of three independent replicates from three different samples of each species. -- not detected.

**Table 4:** Antioxidant activity of the species that were bitten at least twice by sheep during the autumn grazing.

Species	Phenolic Content		Antioxidant Activity		
	Total	Tannins	ABTS^•+ 1^	FRAP ^2^	DPPH^● 3^
	mg Tannic Acid/g		mol Trolox/g	mol Fe^2+^/g	IC_50_ (µg/mL)
*Q. rotundifolia*	309.1 ± 76.99	278.0 ± 77.34	226.5 ± 63.39	100.4 ± 35.53	6.9 ± 3.58
*P. halepensis*	121.3 ± 1.49	108.8 ± 8.61	75.7 ± 14.89	34.9 ± 4.45	18.9 ± 5.09
*P. nigra*	122.3 ± 38.97	102.7 ± 31.78	51.2 ± 19.08	31.8 ± 9.11	26.7 ± 9.13
*J. oxycedrus*	172.7 ± 32.80	132.5 ± 17.00	163.1 ± 47.42	42.7 ± 3.71	16.8 ± 4.90
*J. phoenicea*	138.1 ± 4.15	125.1 ± 1.40	116.3 ± 6.89	40.7 ± 3.66	18.1 ± 0.91
*J. thurifera*	130.1 ± 5.80	93.6 ± 9.01	92.8 ± 5.15	36.8 ± 4.79	23.9 ± 4.19
*B. hirsuta*	65.7 ± 9.02	27.0 ± 8.73	112. 1 ± 36.35	24.7 ± 6.32	55.4 ± 7.07
*G. scorpius*	110.2 ± 7.90	75.6 ± 6.89	41.3 ± 14.75	30.5 ± 1.84	39.4 ± 8.26
*Ph. lychnitis*	93.9 ± 30.92	56.3 ± 34.20	33.5 ± 3.68	38.4 ± 13.05	33.5 ± 10.89
*R. officinalis*	113.6 ± 32.43	84.0 ± 15.06	112.0 ± 36.50	46.0 ± 8.90	17.8 ± 5.52
*S. lavandulifolia*	148.9 ± 23.14	130.3 ± 18.30	36.0 ± 5.19	46.7 ± 5.76	18.8 ± 1.87
*S. leucantha*	132.3 ± 25.49	61.3 ± 11.83	36.5 ± 5.37	46.8 ± 13.18	28.1 ± 3.58
*Th. vulgaris*	162.1 ± 34.10	116.3 ± 8.47	45.3 ± 15.81	59.0 ± 4.11	15.0 ± 4.25
*B. fruticosum*	57.3 ± 5.35	36.3 ± 0.86	21.9 ± 3.65	17.9 ± 3.32	91.0 ± 37.37
*L. fruticosa*	133.4 ± 19.10	66.9 ± 15.98	53.0 ± 18.22	47.7 ± 3.62	16.9 ± 2.34
*P. argentea*	35.4 ± 6.45	20.5 ± 8.56	21.7 ± 8.04	52.2 ± 16.76	141.3 ± 15.13
*H. cinereum*	232.3 ± 64.41	162.7 ± 12.31	220.2 ± 29.24	86.2 ± 2.19	4.6 ± 0.94
*A. humilis*	35.3 ± 5.22	14.3 ± 4.08	21.6 ± 12.12	10.5 ± 1.10	151.3 ± 9.41

^1^ Radical cation decoloration assay; ^2^ ferric reducing antioxidant power; ^3^ radical-scavenging activity. Values are means ± SD of three independent replicates from three different samples of each species.

**Table 5:** Dietary administration of polyphenols present in the concentrate-based diet and in the sage pellets (50% barley-50% distilled *Salvia lavandulifolia* leaves), (mg/g).

Polyphenols	Basal Diet	Basal Diet (10% SBP)
Salvianolic acid	n.d.	0.1 ± 0.02
Daidzin	0.2 ± 0.01	0.2 ± 0.00
Genistin	0.2 ± 0.02	0.2 ± 0.02
Eriodictyol	n.d	0.1 ± 0.00
Genistein	0.1 ± 0.00	0.1 ± 0.00
Caffeic acid	n.d	0.01 ±0.00
Rosmarinic acid	n.d	0.13 ± 0.01
Luteolin	n.d	0.1 ± 0.001
Apigenin	n.d	0.01 ± 0.003
Hispidulin	n.d	0.01 ± 0.002
Diosmetin	n.d	0.04 ± 0.00
Cirsimaritin	n.d	0.1 ± 0.00
Eupatilin	n.d	0.03 ± 0.00
Genkwanin	n.d	0.13 ± 0.00
Salvigenin	n.d	0.1 ± 0.02

Values are means ± SD of three independent replicates. n.d. not detected; SBP: salvia by-products.

**Table 6:** Activities of antioxidant enzymes in ewe and lamb blood (EU/g Hb).

**Ewes**			**SOD**	**GSH-Px**	**CAT**	**SOD/(GSH-Px + CAT)**
Lactation	MG		420 ± 115.47	98 ± 12.19	3771 ± 980.53	0.11
	MI		286 ± 40.55	112 ± 19.03	3918 ± 1046.39	0.07
Weaning	MG		158 ± 27.99	93 ± 19.85	5865 ± 1156.87	0.03 ^a^
	MI		314 ± 70.89	98 ± 15.54	3621 ± 1445.90	0.08 ^b^
**Lambs**						
Lactation	MG		345 ± 147.38	47 ± 13.64	5204 ± 1667.49	0.07 ^a^
	MI		382 ± 147.32	43 ± 18.10	3660 ± 1144.21	0.11 ^b^
Weaning	MG		377 ± 197.96	54 ± 14.79	4345 ± 1373.48	0.09
	MI		309 ± 97.00	51 ± 11.05	4312 ± 1646.95	0.08
Fattening	MG	BDS	303 ± 110.17	56 ± 3.14	7086 ± 2259.87	0.05 ^a^
		BD	226 ± 97.78	59 ± 14.28	4756 ± 1220.68	0.05 ^a^
	MI	BDS	200 ± 39.93	45 ± 9.47	3275 ± 1004.1	0.06 ^a^
		BD	305 ± 158.50	44 ± 19.56	3988 ± 2218.99	0.09 ^b^

MG: mother grazing; MI: mother indoors; BD: basal diet; BDS: basal diet enriched with sage by-products at 10%; SOD: superoxide dismutase; GSH-Px: glutathione peroxidase; CAT: catalase. Within each column (per physiological stage), values followed by different letters show significant differences at 95% (least significant differences).

**Table 7:** Plasma antioxidant status of ewes and lambs (ABTS^• +^, mmol Trolox/mL plasma).

**Ewes**			
Lactation	MG		35.7 ± 2.41 ^a^
	MI		21.5 ± 3.83 ^b^
After lamb weaning	MG		40.7 ± 4.22 ^a^
	MI		30.0 ± 2.62 ^b^
**Lambs**			
Lactation	MG		47.2 ± 2.54
	MI		44.9 ± 4.10
Weaning	MG		42.1 ± 2.72
	MI		42.3 ± 4.32
Fattening	MG	BDS	44.5 ± 4.26 ^bc^
		BD	41.2 ± 1.90 ^ab^
	MI	BDS	47.6 ± 2.87 ^c^
		BD	40.2 ± 2.68 ^a^

MG: mother grazing; MI: mother indoors; BD: basal diet; BDS: basal diet enriched with sage by-products at 10%. Within each column (per physiological stage), values followed by different letters show significant differences at 95% (least significant differences).

**Table 8:** Antioxidant status of lamb muscles.

Tissue	Ewes Rearing System	Diets	FRAP	DPPH^●^	TPC
			mmol Fe^+2^/g	IC_50_ (mg/mL)	µg gallic ac./g
*M. longissimus dorsi*	MG	BD	16.0 ± 2.77 ^c^	65.5 ± 5.84 ^a^	189.7 ± 42.54
		BDS	15.0 ± 3.57 ^b,c^	61.3 ± 5.25 ^a^	181.0 ± 58.91
	MI	BD	11.4 ± 3.15 ^a^	83.4 ± 15.92 ^b^	182.5 ± 33.41
		BDS	12.5 ± 3.41 ^a,b^	69.5 ± 13.85 ^b^	193.3 ± 41.55
*M. deltoideus*	MG	BD	10.8 ± 1.52 ^b^	106.5 ± 5.63 ^c^	211.9 ± 50.25 ^a,b^
		BDS	10.3 ± 1.40 ^b^	76.0± 7.82 ^a^	252.7 ± 47.52 ^b^
	MI	BD	7.6 ± 1.16 ^a^	118.8 ± 22.91 ^c^	190.8 ± 28.71 ^a^
		BDS	9.7 ± 1.65 ^b^	90.5 ± 12.19 ^b^	216.5 ± 27.59 ^a,b^
*M. abdominis*	MG	BD	9.0 ± 1.56	89.1 ± 6.68	118.6 ± 28.95 ^a^
		BDS	9.1 ± 1.93	93.0 ± 13.93	151.2 ± 23.24 ^a^
	MI	BD	9.6 ± 1.69	84.6 ± 7.67	221.7 ± 34.71 ^b^
		BDS	9.1 ± 1.98	92.3 ± 12.68	199.9 ± 34.34 ^b^

MG: mother grazing; MI: mother indoors; BD: basal diet; BDS: basal diet enriched with sage by-products at 10%; FRAP: ferric reducing antioxidant power; DPPH^●^: radical-scavenging activity; TPC: total phenolic content. Within each column (per tissue), values followed by different letters show significant differences at 95% (least significant differences).

## Supplementary Materials

The following are available online at https://www.mdpi.com/2076-3921/9/10/938/s1, Table S1: Chemical composition of the basal lamb diet.
