# Supplementary material for: The Combined Effect of Mediterranean Shrubland Pasture and the Dietary Administration of Sage By-Products on the Antioxidant Status of Segureña Ewes and Lambs
Source: Antioxidants (Basel). 2020 Sep 30;9(10):938. doi: 10.3390/antiox9100938 (PMC7600900; doi:10.3390/antiox9100938)
Supplement: Supplementary file 1 [file antioxidants-09-00938-s001.pdf]

Table S1. Chemical composition of the basal lamb diet.

Analitical Components	Concentrate [a] g/Kg Dry Matter
Ash	63.2
Crude Protein (CP)	170.0
Fat	37.5
Neutral Detergent Fiber (NDF)	366.6
Digestible (NDF) (%)	25.2
Acid detergent fiber	191.3
Nonproteic nitrogen	0.5
Ruminal degradable protein (RDP), % of CP	65.4
Nonfiber carbohydrate	268.3
Adjusted total starch	91.4
Total soluble RDP	43.1
Ruminal undegradable protein	56.3
Calcium	8.3
Phosphorous	4.1
Sodium	2.6
Magnesium	2.0
**Additives**	
Vitamin A (IU/Kg)	10.000.000
Vitamin D3 (IU/Kg)	2.000.000
Vitamin E (mg/kg)	30
Manganese (mg/kg)	37
Iron (mg/kg)	15
Copper (mg/kg)	8
Zinc (mg/kg)	50
Iodine (mg/kg)	0.5
Cobalt (mg/kg)	0.05
Selenium (mg/kg)	0.4
Antioxidants (mg/Kg)	
Ethoxyquin	1.8
Butylated hydroxyanisole	1.35
Propyl gallate	0.37

Formulated using the following ingredients g/Kg: Corn 285; barley 150; Wheat 240; Soybean meal 163; Distiller's dried grains with solubles 70; Wheat bran 30; Sugarcane molasses 20; Soy crude oil 5: Calcium carbonate 17.7; Sodium chloride 5; Other minerals 10.5. Data provided by Alimer (Alimentos del Mediterráneo, Lorca, Murcia, Spain).
